# Frontal lobe regulation of blood glucose levels: support for the limited capacity model in hostile violence-prone men

**DOI:** 10.1007/s40708-016-0034-6

**Published:** 2016-02-01

**Authors:** Robert P. Walters, Patti Kelly Harrison, Ransom W. Campbell, David W. Harrison

**Affiliations:** Neuroscience Laboratory, Department of Psychology, Virginia Polytechnic Institute & State University, Williams Hall, Virginia Tech, Blacksburg, VA 24061 USA

**Keywords:** Hostility, Aggression, Diabetes mellitus, Glucose, Metabolic syndrome, Cardiovascular

## Abstract

Hostile men have reliably displayed an exaggerated sympathetic stress response across multiple experimental settings, with cardiovascular reactivity for blood pressure and heart rate concurrent with lateralized right frontal lobe stress (Trajanoski et al., in Diabetes Care 19(12):1412–1415, 1996; see Heilman et al., in J Neurol Neurosurg Psychiatry 38(1):69–72, 1975). The current experiment examined frontal lobe regulatory control of glucose in high and low hostile men with concurrent left frontal lobe (Control Oral Word Association Test [verbal]) or right frontal lobe (Ruff Figural Fluency Test [nonverbal]) stress. A significant interaction was found for Group × Condition, *F* (1,22) = 4.16, *p* ≤ .05 with glucose levels (mg/dl) of high hostile men significantly elevated as a function of the right frontal stressor (*M* = 101.37, SD = 13.75) when compared to the verbal stressor (*M* = 95.79, SD = 11.20). Glucose levels in the low hostile group remained stable for both types of stress. High hostile men made significantly more errors on the right frontal but not the left frontal stressor (*M* = 17.18, SD = 19.88) when compared to the low hostile men (*M* = 5.81, SD = 4.33). These findings support our existing frontal capacity model of hostility (Iribarren et al., in J Am Med Assoc 17(19):2546–2551, 2000; McCrimmon et al., in Physiol Behav 67(1):35–39, 1999; Brunner et al., in Diabetes Care 21(4):585–590, 1998), extending the role of the right frontal lobe to regulatory control over glucose mobilization.

## Introduction

A key feature of hostility is the exaggerated and prolonged stress response that has been implicated in the development of cardiovascular disease [1–3], hypertension [4, 5] atherosclerosis [6], and death [1]. Traditionally, the stress response of hostile individuals has been examined using cardiovascular measures. However, the mechanisms underlying these disease processes may reside in the stress-related products of glucose, lipids, and cholesterol that mobilize as the body readies itself for action. Moreover, the literature on hostility and/or anger supports variant levels of glucose ADA [7–11], lipids [12, 13], and cholesterol [14–16] in these individuals that are prone to develop cardiovascular disease. Poor regulatory control of these stress-related processes may implicate diminished frontal capacity and especially within the right cerebral system regulating anger.

### Defining anger and anger expression

Prior to discussing hostility and anger-related problems, the construct must be clearly defined. Definitions of these constructs often vary as some view them as distinct and others discuss them as components of a unitary construct with violence-prone behavior drawn from a hostile and cynical view toward others. For the purposes of the current research, hostility was operationally defined from among these multidimensional constructs with distinct affective, behavioral, and cognitive dimensions and distinct physiological elements that contribute to both the experience and expression of the emotion [17]. The affective dimension of anger refers to the emotional state that occurs in response to an immediate stressor and may vary in both intensity and duration [18]. The cognitive dimension of anger, also referred to as hostility in the literature, has most frequently been defined as a cognitive phenomenon of an attitudinal nature that subserves the emotional process but is not an emotion *per se* [19]. The behavioral dimension of anger is simply the behavioral response to the subjective experience of anger [20] and may be expressed outwardly or inwardly [18].

Despite the documented association between these constructs, controversy remains over the level of these products in relation to hostility (i.e., too much or too little). In addition, there are very few models of hostility that attempt to explain how these constructs are related. To address these concerns, *The Limited Capacity Model of Hostility* was proposed by Williamson and Harrison [21], Carmona et al. [22], and Mitchell and Harrison [23]. Specifically, we have proposed a limitation in capacity of the right frontal lobe to regulate posterior and inferior cerebral systems under stress. To test this model, blood glucose mobilization was recorded using a pre–post-stress paradigm. However, unlike traditional stress research, this experiment employed lateralized left and right frontal lobe stressors using verbal or nonverbal fluency tasks.

### Hostility and metabolic factors

The stress response in hostile men has been measured with cardiovascular indices; however, additional metabolic factors have been employed as markers of heightened levels of arousal. Vogele [13] found high and low hostile men to have differing lipid levels at baseline. After an overnight fast, the hostile men had higher triglyceride levels and very low-density lipoproteins (VLDL) when compared to the low hostile men. In addition to the differences at baseline, Finney et al. [12] reported that men with elevated levels of anger displayed increased lipids and blood pressure after a stress condition. Specifically, hostile men demonstrated lipid reactivity subsequent to a speech stressor relative to low hostile men. Cholesterol has proven to be more controversial with numerous contradictory results. Richards et al. [16] found that the participants with elevated scores on hostility and aggression measures also had increased cholesterol levels. In a sample of hospitalized men with a history of violent behavior, cholesterol levels were found to be lower than the general population [15]. Despite previous associations between cholesterol and hostility, Fowkes et al. [14] demonstrated no relationship between the two. Regardless, metabolic factors remain most relevant as an indicator of stress in hostile men.

### Glucose

This experiment examined the role of glucose in a hostile population. The rational for this selection is that glucose is an integral fuel source for the brain and despite only consisting of 2 % of an individual’s total mass, the brain consumes almost 50 % of the available glucose [24]. Irregularities in glucose levels, such as hyperglycemia (blood glucose over 120 mg/dl) or hypoglycemia (blood glucose under 70 mg/dl) (ADA) have resulted in increased hostile and aggressive behaviors [7, 8, 10, 11]. Despite this association, a point of contention remains as to whether it is the hypoglycemic or hyperglycemic episodes that are responsible for the increased levels of anger, hostility, and aggression. In addition, there has been no known research examining the role of glucose and hostility from a neuropsychological perspective.

The ADA asserts that a key group of indicators in an individual having a hypoglycemic episode is the “autonomic symptoms” (American Diabetes Association Complete Guide to Diabetes, [25]. These include the opening of blood vessels, increased blood pressure, increased heart rate, as well as fluctuations in emotional states to include increased anger. The ADA also affirms that prolonged hypoglycemic episodes have been associated with heart disease, because individuals with hypoglycemia have increased heart rates for extended periods. Aside from these autonomic symptoms, the ADA describes the effect of low blood sugar on the brain to include anger, lack of coordination, confusion, personality change, and unconsciousness, among others (p. 161). High hostile men have also reliably reported a lack of awareness of their hostility classification [26, 27] compared to low hostile men. This shared lack of self-awareness, in those with heightened levels of hostility and those with decreased levels of blood glucose, may be related to diminished right frontal function.

Virkkunen [11] examined the role of hypoglycemia in violent offenders using a glucose tolerance test, finding habitually violent offenders to have hypoglycemic tendencies when compared to non-violent offenders. Benton et al. [7] documented increased aggression after an induced hypoglycemic episode. After fasting for nearly 12 h, the men had increased levels of aggression as measured by multiple aggression measures including the Cook-Medley Hostility Scale (CMHO). Employing the glucose clamp technique, McCrimmon et al. [8] reported that participants had increased levels of anger and frustration on the State-Trait Anger Expression Inventory (STAXI) after the hypoglycemic episode. Donhoe and Benton [28] found similar results with nondiabetic women. After having participants fast overnight, the researchers administered an oral glucose tolerance test. Lower blood glucose levels were associated with increased scores of aggression and frustration in the Rosenzweig Picture-Frustration Study.

In contrast to hypoglycemia, evidence exists for hostility’s role in hyperglycemia. Raiikkonen, et al., [9] examined the influence of psychosocial variables on the Insulin Resistance Syndrome (IRS) concluding that hostility, among other constructs, was associated with the variables of hyperinsulinemia, hyperglycemia, dyslipidemia, hypertension, and increased abdominal adipose tissue among others. These associations between hostility and metabolic disturbances were found in healthy, middle-aged men employed as managers after a 12-h fast and were argued to demonstrate the effects of personality, behavioral patterns, and a stress-inducing lifestyle on insulin resistance.

As part of the Normative Aging Study, Niaura et al. [29] reported that hostility was positively associated with fasting insulin level and a number of other metabolic factors. Here, subjects were initially enrolled in 1986 and followed thereafter. High scores on the CMHO were positively associated not only with decreased insulin levels, but also with waist/hip ratio, body mass index, total caloric intake, and serum triglycerides. Path analyses revealed that the effects of hostility on insulin, triglycerides, and high-density lipoprotein cholesterol were mediated by body mass index.

The relationship between hostility and heightened levels of glucose may be evident even in childhood and adolescence. In a sample of 134 African American and European American children, Raikkonen et al. [30] found that baseline hostility scores on the CMHO predicted future metabolic syndrome diagnoses for children and adolescences that did not have the metabolic syndrome at baseline at the time of a 3-year follow-up. The authors suggested that insulin resistance and obesity were primarily responsible for the relationship between hostility and the metabolic syndrome.

### The neuropsychology of glucose levels in the hostile population

Despite the documentation of the relationship between hostility and glucose irregularities, the literature falls short when providing a theory to explain this connection. From a neuropsychological perspective, it is argued that altered functioning in the right hemisphere may be responsible. The right hemisphere has long been implicated in the processing of emotion [31–33]. Our laboratory has provided evidence of four primary quadrants (anterior–posterior and left–right cerebral hemispheres) contributing to emotional processing [34–38]. Specifically, we have demonstrated increased arousal for right hemispheric auditory [26], visual [39, 40], vestibular [22], and somatosensory processing [41, 42]. Moreover, hostile men have shown evidence for diminished capacity within right anterior cerebral regions, including motor [43] and premotor systems [21, 44–46]. Collectively, this approach has culminated in the *Limited Capacity Model* [21–23, 35, 46]. We proposed that anger regulation and concurrent regulatory control over sympathetic drive suffer in hostile, violent-prone men due to diminished capacity within right frontal systems.

In a test of this model, Williamson and Harrison [21] investigated the left and right prefrontal regions in a hostile population when evaluating cardiovascular reactivity to lateralized prefrontal stressors. The Controlled Oral Word Association Test (COWAT) and Ruff Figural Fluency Test (RFFT) were used as verbal and nonverbal frontal lobe stressors. Previous research has demonstrated the COWAT to be sensitive to left frontal functioning [47], whereas the RFFT is sensitive to right frontal functioning [26, 44]. The results indicated that the verbal and nonverbal stressor tests produced diametrically opposite effects on systolic blood pressure in high hostile males. Specifically, systolic blood pressure increased subsequent to the right frontal stressor, whereas systolic blood pressure decreased subsequent to the left frontal stressor. This research has implications for the role of the left and right frontal regions in cardiovascular regulation in hostile men. Williamson and Harrison [21] concluded that the right frontal regions were unable to regulate sympathetic tone with the concurrent demand of the lateralized stressor task proposing *The Limited Capacity Model*. This research is in accord with, and extends, previous research on the anterior–posterior model of anger regulation, specifically supporting diminished right frontal capacity in hostile men. Diminished capacity within the right frontal region may be expressed in poor regulatory control over anger and hostility and over sympathetic drive.

Hostility, a personality trait characterized by the increased experience of negative emotion, is conveyed through heightened sympathetic arousal, as measured through increases in heart rate and systolic blood pressure. Glucose levels in hostile men have yet to be examined despite the key role of glucose in response to threat or provocative negative emotional stress. Unlike other arousal mechanisms, there is a finite amount of glucose in the body, and the brain requires specific levels to function at an optimal level. Under right frontal stress, glucose levels should increase dramatically with sympathetic activation. Moreover, hostile men may poorly regulate glucose mobilization because of deficient right frontal capacity for regulatory control over the sympathetic arousal response.

### Hypotheses

1. High hostile men will have increased sympathetic arousal, as measured by glucose, as a function of the right frontal stressor (RFFT) and will have decreased responses to the left frontal stressor (COWAT).
2. High hostiles will have lower performance scores on the right frontal stressor.

## Method

### Participants

One hundred and fifty one men completed the online screening in return for extra credit in their undergraduate psychology courses. Participants were initially screened online using the Cook-Medley Hostility Scale (CHMO). High hostile participants were defined as those scoring 28 or above on the CHMO (maximum score = 50). Low hostile participants were defined as those scoring 19 or below on the CHMO. These cut-off scores represent the upper and lower thirds of the CHMO distribution and are consistent with previous research on hostility [26, 39, 43]. From this initial screening, 34 right-handed men met criteria as either low or high hostile men and agreed to participate in the experiment. Women participants were not included at any point of the online screening or the experiment due to sex differences in cerebral laterality [48–50]. Overall, the participants reported no previous history of developmental problems, hypoglycemia, hyperglycemia, hypertension, hypotension, or hyperthyroidism. Participants reported no history of head injury, loss of consciousness for more than 5 min, neurological, or psychiatric disorder, heart disease or pancreatic disease. Participants were not currently taking allergy or “illegal” medications. Participants were excluded if they were smokers of tobacco products or if they consumed three or more drinks of alcohol more than twice a week.

Using these criteria, two participants were excluded based on their responses on the Medical History Questionnaire. Four participants were excluded because of variant scores on their second completion of the CMHO, which occurred in the laboratory to ensure stable hostility levels. One participant’s score on the CMHO changed by 10 points, resulting in a reversal of his group inclusion from low hostile to high hostile. Three additional participants scores regressed toward the mean and did not meet criteria for either the low or the high hostile group. Finally, two participants from each group (high and low) were excluded due to extreme scores on the second measurement of the CMHO. Thus, 24 healthy, right-handed, nonsmoking men participated in the experiment. Specifically, 12 high hostile and 12 low hostile men participated in the project.

### Self-report measures

The 50-item Cook-Medley Hostility Scale (CMHO) has been frequently used as a valid predictor of hostility [51, 52]. Originally based on portions of the Minnesota Multiphasic Personality Inventory [10], the CMHO is the most commonly used hostility measure and is a valid predictor of medical, psychological, and interpersonal outcomes of trait-based hostility [53]. According to Christensen et al. [54], the CMHO has proven to have reliable internal consistency (coefficient alpha *r* = .86). Test–retest consistency confirmation is also reliable (*r* = .84).

Handedness or hemibody preference was determined using scores on the Coren, Porac, and Duncan Laterality Questionnaire (CPDL; [55]. Only right-handed subjects were used scoring +7 or above on this instrument.

Subjects also completed a Medical History Questionnaire from our laboratory to insure that they had not been diagnosed with significant medical or psychiatric problems, including head injury.

### Blood glucose measurement

The current research on glucose measurement indicates marked benefits from obtaining glucose from the forearm [56–58]. The Therasense Freestyle Glucometer is a leading device for forearm testing [59]. In comparison to the One Touch, Ultra Blood Glucose Monitoring System, The Freestyle Glucometer maintains increased accuracy, demonstrates more clinically acceptable readings when compared to intravenous blood samples, and requires fewer sticks [60].

There is much controversy concerning the at-home, self-test measurement of blood glucose levels, and all of the glucometers assessed to date, have failed to meet the 95 % accuracy rating standard set by the ADA [61–63]. Historically, manufacturers of home glucose monitoring devices have recommended obtaining blood samples from the fingertips to assess blood sugar levels. There is some controversy over the accuracy of forearm testing particularly concerning the difference between forearm and finger sites when glucose levels are rapidly ascending or descending. Peled et al. [64] had participants sugar load and found the forearm testing to be less accurate at detecting the swift change in glucose levels when compared to the finger tip sites, yet found the forearm to be reliable, otherwise. Lee et al. [56, 57] demonstrated a few significant differences in the level of accuracy after employing the two methods for 190 diabetics over the course of the day. Other researchers have found no difference between finger-prick testing and forearm measurements even with rapid changes in participants’ glucose levels [65]. Regardless of the controversies, a marked benefit in forearm testing has been the ease of obtaining a blood sample and the noteworthy decrease in pain [56–58]. Forearm testing further increases the readiness for testing, particularly when frequent blood samples are required.

### Behavioral measures

#### Verbal stressor (verbal fluency)

The Controlled Oral Word Association Test (COWAT) is a measure of verbal fluency [47]. The COWAT consists of three one-minute trials in which participants are instructed either to say as many words that begin with a specific letter as possible. Proper names, numbers, and the same word with a different suffix do not qualify. These responses, scored as errors, are subtracted from the total number of words generated on the test. In accordance with previous research [21, 66], the letters F, S, and T were used based on the tendency for the normal population to produce nearly equal words for each letter (10–12 per min).

Individuals with left frontal lobe deficits often have lower scores on this verbal fluency test when compared to a normal population [67, 68]. In addition, individuals with lesions in the left frontal lobe have decreased performance when compared to individuals with right frontal lobe lesions [69, 70].

#### Nonverbal stressor (nonverbal fluency)

The Ruff Figural Fluency Test (RFFT) is a paper and pencil test consisting of five sections used as a measure of nonverbal fluency. Within each section, there are 35 dot matrices arranged in a 5″ × 7″ pattern. The participants had 1 min to connect three or more dots, making as many unique patterns as possible in the time allotted. Scoring consists of counting the number of patterns minus the number of perseverative errors for each trial. The total score is the number of patterns produced minus the number of perseverative errors. A perseverative error consists of a repetition of a design. In accordance with previous research [21, 66], three sheets containing the 35 dot matrices instead of five sheets were used to maintain uniformity with the COWAT. Scores were totaled across trials.

The RFFT is thought to be a measure of right frontal lobe functioning. Previous research has demonstrated that individuals with right frontal lobe strokes or brain injuries have significantly lower scores, or increased error ratios on nonverbal fluency tasks, compared to those without right frontal lobe deficits [71]. More recently, Foster et al. [44] demonstrated the significant relationship between performance on the RFFT and right frontal capacity in healthy young adults. More specifically, the low design fluency group evidenced increased delta magnitude over the right frontal region using quantitative electroencephalography.

#### Procedure

After completion of the CHMO, participants meeting the criteria for either low or high hostility were contacted for completion of the next phase of the experiment. Subsequent to review of the completed online Medical History Questionnaire, participants were given a brief outline of the experiment and informed that a forearm prick would be administered. Before entering the laboratory, participants were requested to abstain from caffeine, tobacco, and alcohol and to eat a small meal or snack.

Upon arrival at the laboratory, the participant reviewed and signed the Informed Consent Form. The CMHO was completed again to ensure stability of the hostility scores. The researcher left the room and repeated the following instructions: “Please take about 1 min to become accustomed to your surroundings. Please sit still in the chair and face forward.” After a 90-s adaptation period, the experimenter reentered the room and recorded baseline measures of glucose. Blood glucose was measured at the left forearm, which was cleaned with an alcohol swab and then quickly lanced.

Immediately after recording baseline levels of glucose, participants were instructed that they would complete either the verbal or the nonverbal fluency measure, the order of which was counterbalanced for each participant entering the laboratory. Subjects were instructed on the fluency measure and the task was completed. Glucose levels were assessed again following the completion of the task. Following a 90-s adaptation period, glucose levels were recorded again and the second task was administered. Glucose levels were recorded immediately after the second task.

## Results

### Self-report measures

Test-rest reliability of the CMHO yielded a value of *r* = .95. This is higher than the *r* = .84 reported by Christensen et al. [54]. The range for this measure was 30 with a low score of 8 and a high score of 38.

### Physiological measures

A 3-way mixed design Analysis of Variance (ANOVA) was performed on the variable of glucose level (mg/dl), with the fixed factor of Group (high and low hostile) and with repeated measures for Stress Condition (verbal and nonverbal stressor) and Trial (pre- and post-stress). Post hoc comparisons were made using Tukey’s LSD [72]. An a priori level of significance was set at *p* ≤ .05.

**Fig. 1:**
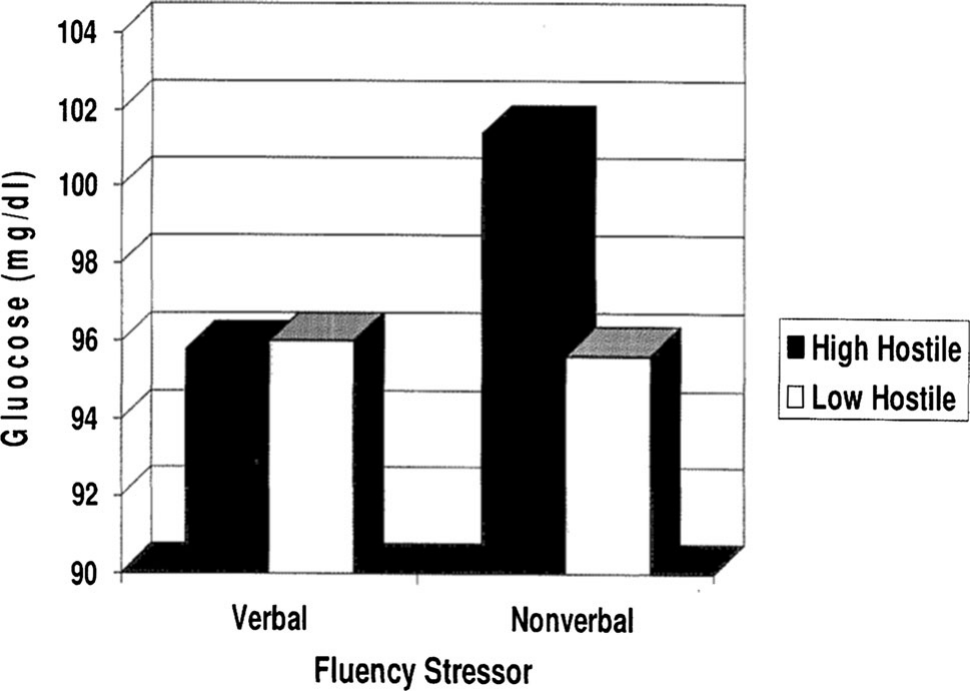
Group differences in Glucose levels (mg/dl) at a function of Condition (verbal or nonverbal stressor)

High hostile men were expected to have increased physiological arousal as measured by glucose as a function of the nonverbal stressor and decreased physiological arousal to the verbal stressor. A significant interaction was found for Group × Condition, *F* (1,22) = 4.16, *p* ≤ .05. For the high hostile group, glucose levels (mg/dl) were significantly higher for the nonverbal stressor (*M* = 101.37, SD = 13.75) when compared to the verbal stressor (*M* = 95.79, SD = 11.20). For the low hostile group, glucose levels (mg/dl) remained stable, or unchanged, as a function of the nonverbal stressor (*M* = 95.63, SD = 22.04) and as a function of the verbal stressor (*M* = 96, SD = 21.34). Group differences in mean glucose levels as a function of Condition can be seen in Fig. 1. The interaction effect of Group × Condition × Trial for glucose was not significant, *F* (1,22) = .53, *p* *>* .47.

### Behavioral measures

**Fig. 2:**
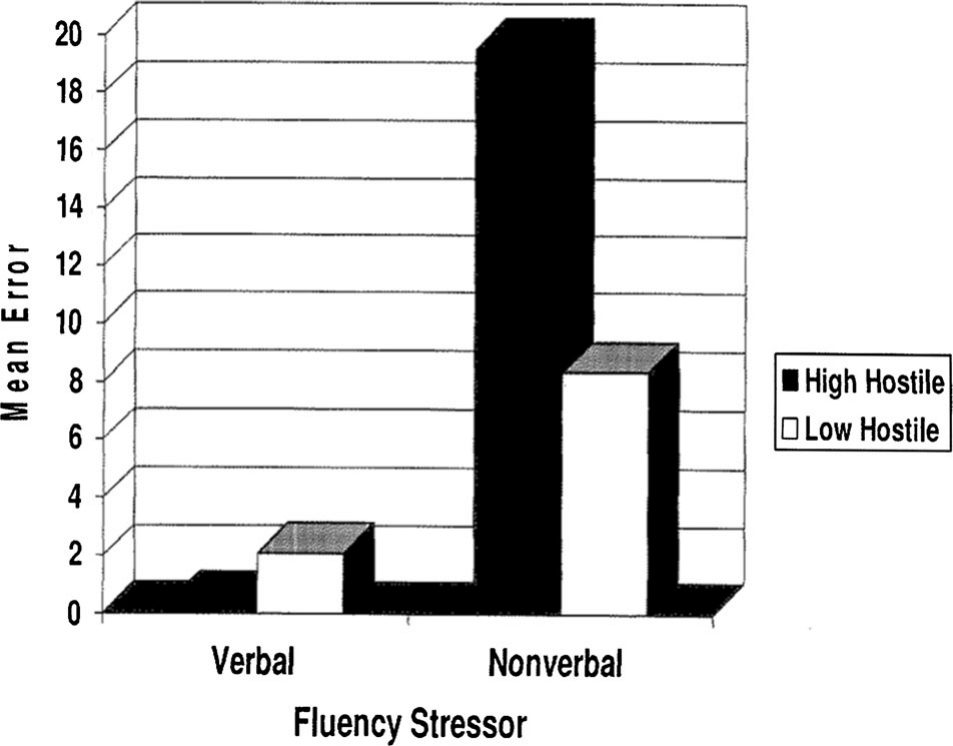
Mean error score as a function of Group and Condition (verbal or nonverbal stressor)

For the behavioral measures, total fluency scores and total error scores were analyzed using separate 2-way mixed design ANOVAs with the fixed effects of Group (high and low hostile) and with the repeated measures of Condition (verbal and nonverbal stressors). It was predicted that group classification would affect performance on the verbal and nonverbal stressors. Specifically, it was predicted that the high hostile men would have lower scores on the nonverbal stressor (RFFT) than on the verbal stressor (COWAT) in both within group and between group comparisons. There was partial support for this prediction as a significant interaction effect was found for Group × Condition, *F* (1,22) = 4.90, *p* *>* .03. The high hostile men made significantly more errors on the nonverbal stressor (*M* = 17.18, SD = 19.88) when compared to the low hostile men (*M* = 5.81, SD = 4.33). On the verbal stressor, the high hostile men made significantly fewer errors (*M* = .04, SD = 0.66) when compared to the low hostile group (*M* = 2.08, SD = 2.93) (see Fig. 2). It should noted that due to the extreme variability in error scores (range = 53, SD = 14.05, Variance = 197.56, CV = 176.92) the outliers (1) in each group were excluded.

However, no significant interactions were found for the additional analyses of the behavioral measures to include the variable of total fluency score. The interaction of Group × Condition, *F*(1,22) = .59, *p* ≤ .59 for total fluency score was not reliable and reflected no difference among groups for the number of correct items on the verbal and the nonverbal stressors.

## Discussion

The literature on hostility is robust with findings from multiple areas within psychology, which reflect both the complexity and the evolution of the construct over time [73, 74]. In accord with previous research on hostility evaluating cardiovascular deregulation in hostile men using left and right frontal fluency stress [21, 75], the present experiment employed lateralized stressors in this population with a diminished capacity for negative emotional regulation. However, the current experiment extended this earlier research by measuring blood glucose mobilization as a function of concurrent left and right frontal lobe stressors in high and low hostile men.

Two primary findings from the current experiment add to the existing hostility literature. The first is that high and low hostiles mobilize glucose at different rates as a function of lateralized frontal stressors. Specifically, high hostile men mobilize heightened levels of glucose to nonverbal stress when compared to both verbal stress and to low hostiles. Further, the glucose levels of the low hostile men remain stable despite the completion of the left and the right frontal stressors. These findings potentially support the interpretation of limited right frontal capacity in hostile men with diminished regulatory control over anger and cardiovascular function, whereas the present results implicate poorly regulated blood glucose levels in this group under right frontal lobe stress.

The second major finding is that high hostiles make more errors on a design fluency task, when compared to the verbal fluency task and to the errors made by the low hostile group. This finding indicates that high hostile men have difficulty manipulating spatial arrangements under a time constraint. Moreover, the results support the limited right frontal lobe capacity interpretation, where high hostiles deregulate glucose when confronted with a dual task challenge for the right frontal region. The results support increased frontal regulatory capacity among low hostiles where there is glucose stability with verbal or nonverbal stressors and where performance on these measures is superior to the high hostile men.

The findings from this experiment support a right hemispheric model of hostility. Here, hostility has been previously associated with increased activation for auditory [26], visual [39, 40], vestibular [22], and somatosensory modalities [41, 42]. Diminished regulatory capacity of the right frontal regions has received further support from investigations of motor [43] and premotor systems [21]. Within this model, high hostile men have a diminished capacity for concurrently completing a right frontal lobe stressor, while inhibiting or regulating sympathetic systems. Thus, right frontal stress results in the increased activation and exaggerated responses for cardiovascular systems [21–23] and altered sensory and perceptual appraisal of emotional stimuli across modalities.

Previous research on emotion has focused on the negative valences, including hostility, from a functional cerebral systems perspective to determine neuropsychological evidence of laterality effects and of regulatory control mechanisms. Shapiro et al. [76] used single-photon emission computed tomography (SPECT) to measure cerebral blood flow following administration of a stressor (mental arithmetic) to hostile men. These researchers demonstrated that the stressor decreased prefrontal blood flow in the left frontal-temporal regions in the high hostile group. The high hostile group also showed marginal increases in heart rate during the stressor. The authors did not appreciate the relative right cerebral deactivation associated with increased heart rate. Shapiro et al. [76] conclude that the prefrontal regions may regulate cardiac changes. Moreover, hostility may exacerbate these conditions with characteristic features of deregulation and reactivity to stress.

In efforts to establish the cerebral mechanisms responsible for hostility, Louis et al. [77] administered PET scans to 10 normal adult men (mean age = 25). The PET scans were purported to assess ongoing metabolic processes and to provide a more direct means of localizing cerebral metabolic glucose rates. After the infusion of D-[F] deoxyglucose (FDG), participants reported their thoughts, feelings, and free associations, which were blindly scored using the Gottschalk-Gleser Anxiety and Hostility Scale with 90 words or more being the criterion for a reliable sample. Examination of the white matter revealed significant positive correlations between hostility and glucose metabolic rates at the right superior frontal, the right superior parietal, and the right occipital lobes. Heightened metabolic rates provided supportive evidence of right cerebral activation with hostility.

Two potential explanations are offered in the interpretation of these results. Initially, the high hostiles’ increase in glucose levels to the nonverbal stressor provides evidence for a faulty system with a diminished capacity for regulation of the appropriate glucose levels. In accord with Kinsbourne’s Functional Cerebral Space Model [78, 79], the completion of the nonverbal stress by the high hostiles produced an interference effect with the regulation of the sympathetic nervous system [21, 22]. This interference effect is also evident in the increased mobilization of glucose as the high hostiles have a diminished capacity to concurrently regulate both systems. It follows that the over-appraisal of negative affect among those exhibiting emotional lability for anger would occur with physiological responses in preparing for “fight” or “flight” to include glucose mobilization, as demonstrated here, and potentially cholesterol and other substances, which negatively affect long-term health and cardiovascular disease.

A second interpretation of these findings may support Selye’s [80] model of stress whereby the stress that is applied to a system causes a response to remove the stress and to return to ‘pre-stress’ levels. Applying this concept to the current experiment provides evidence for the diminished capacity of the high hostiles in regulating their stress response albeit a stress response regulated by right frontal systems that play a role in anger modulation and mobilization for the sympathetic nervous system.

It may also be the case that the high hostiles require this influx of fuel to cope with stress, or in the case of this experiment, to complete the right frontal stressor. The high hostiles may potentially be metabolizing glucose at a greater rate to compensate for regions with diminished capacity, specifically the right anterior cites. Dwyer [81] who reports that glucose is the primary fuel for the brain and that this substance is involved in nearly all of the brain’s activities, to include all cognitive abilities and nearly all cellular processes, finds support for this. Dwyer further notes that the regulation of glucose is not fully understood, especially at a global level, however, glucose dysregulation has been associated with depression [82] and schizophrenia [83] as well as diabetic and hypoglycemic conditions that underlie cardiovascular disease.

Global changes are evident in those with diabetes. In a review of the literature on glucose, McCall [84] finds that those with diabetes have between a two-and a six-fold risk of experiencing a stroke. Further, those surviving a stroke will have greater difficulty with recovery, as neurotransmitter metabolism is altered. Interestingly, McCall sites Woo et al. [85] who reports that the increased risk of stroke in diabetics may be the result of the stress response. Specifically, when diabetics are experiencing a hyperglycemic episode, they continue to mobilize glucose in response to stress, thereby further increasing their glucose levels, and eventually resulting in a vascular accident. It appears as if glucose regulatory dysfunction often leads to heightened dysfunction subsequent to stress. From a functional cerebral systems view, it appears that the right frontal region in particular is unable to inhibit a reflex glucose release, resulting in continued glucose mobilization and instability in the associated affective, sympathetic, and cognitive processing systems.

Although there may be additional mechanisms linking glucose dysfunction and hostility, it appears as if both variables are involved in the stress response. Unfortunately, the long-term consequences of an exaggerated stress response, as experienced by those with heightened levels of hostility, can be deadly. Previously, hostility has been linked to heart disease, cardiovascular disease, hardening of the arteries [51, 86, 87], altered cholesterol levels [88, 12], lipid dysregulation [13], and most notably glucose dysregulation [8, 10, 28]. Interestingly, as part of the Atherosclerosis Risk in Communities (ARIC) study researchers examined over 6000 participants and reported that glucose and heart function are strongly intertwined. The presence of one of these factors increases the likelihood of the other. The report ultimately states that the researchers are unsure how changes in glucose levels and changes in heart function are related. However, those individuals with cardiac problems have a grim prognosis if glucose dysregulation is present [89].

The present experiment provides for evidence of functional neural systems differentially responding to verbal as opposed to nonverbal figural fluency stressors. The project sets within a line of systematic research on the hostility construct, anger, and violence-prone behavior (see [90]. However, the present project remains limited due to the small sample sizes and potentially from the limited statistical approach, where the groups were identified using cut-off scores rather than a continuous measure.

## References

[1] Boyle SH, Williams RB, Mark DB, Brummett BH, Siegler IC, Helms MJ, Barefoot JC (2004). Hostility as a predictor of survival in patients with coronary artery disease. Psychosom Med.

[2] Brydon L, Magid K, Steptoe A (2005). Platelets, coronary heart disease, and stress.

[3] Miller TQ, Smith TW, Turner CW, Guijarro ML, Hallet AJ (1996). A meta analytic review of research on hostility and physical health. Psychol Bull.

[4] Yan LL, Liu K, Mathews KA, Daviglus ML, Ferguson TF, Kiefe CI (2003). Psychosocial factors and risk of hypertension: the coronary arty risk development in young adults (CARDIA) study. J Am Med Assoc.

[5] Zhu H, Poole J, Lu Y, Harshfield GA, Treiber FA, Snieder H, Dong Y (2005). Sympathetic nervous system, genes and human essential hypertension. Curr Neurovasc Res.

[6] Iribarren C, Sidney S, Bild DE, Liu K, Markovitz JH, Roseman JM, Mathews K (2000). Association of hostility with coronary artery calcification in young adults: the CARDIA study. J Am Med Assoc.

[7] Benton D, Kumari K, Brain PF (1982). Mild hypoglycemia and questionnaire measures of aggression. Biol Psychol.

[8] McCrimmon RJ, Ewing FME, Frier BM, Deary IJ (1999). Anger state during acute insulin-induced hypoglycemia. Physiol Behav.

[9] Raiikkonen K, Keltikangas-Jarvinen L, Adlercreutz H, Hautanen H (1996). Psychosocial stress and the insulin resistance syndrome. Metabolism.

[10] Surwit RS, Williams RB, Siegler IC, Lane JD, Helms M, Applegate KL, Zucker N, Feinglos MN, McCaskill CM, Barefoot JC (2002). Hostility, race, and glucose metabolism in nondiabetic individuals. Diabetes Care.

[11] Virkkunen R (1982). Reactive hypoglycemic tendency among habitually violent offenders. A further study by means of the glucose tolerance test. Neuropsychobiology.

[12] Finney ML, Stoney CM, Engebretson TO (2002). Hostility and anger expression in African-American and European American men is associated with cardiovascular and lipid reactivity. Psychophysiology.

[13] Vogele C (1997). Serum lipid concentrations, hostility, and cardiovascular reactions to mental stress. Int J Psychophysiol.

[14] Fowkes FG, Leng GC, Donnan PT, Deary IJ, Reimersma RA, Housley E (1992). Serum cholesterol, triglycerides, and aggression in the general population. Lancet.

[15] Hillbrand M, Waite BM, Miller DS, Spitz RT, Lingswiler VM (2000). Serum cholesterol concentrations and mood states in violent psychiatric patients: an experience sampling study. J Behav Med.

[16] Richards JC, Hof A, Alvarenga M (2000). Serum lipids and their relationships with hostility and angry affect and behaviors in men. Health Psychol.

[17] Spielberger CD, Johnson EH, Russell SF, Crane RJ, Jacobs GA, Worden TJ, Chesney MA, Rosenman RH (1985). The experience and expression of anger: Construction and validation of an anger expression scale. Anger and hostility in cardiovascular and behavioral disorders.

[18] Spielberger CD, Reheiser EC, Sydeman SJ, Kassinove H (1995). Measuring the experience, expression, and control of anger. Anger disorders: definitions, diagnosis, and treatment.

[19] Smith TW, Goreczny AJ (1995). Assessment and Modification of Coronary-Prone Behavior. Handbook of health and rehabilitation psychology.

[20] Weiner B (1995). Judgments of responsibility: a foundation for a theory of social conduct.

[21] Williamson JB, Harrison DW (2003). Functional cerebral asymmetry in hostility: a dual task approach with fluency and cardiovascular regulation. Brain Cogn.

[22] Carmona JE, Holland AK, Harrison DW (2009). Extending the functional cerebral systems theory of emotion to the vestibular modality: a systematic and integrative approach. Psychol Bull.

[23] Mitchell GA, Harrison DW (2009). Neuropsychological effects of hostility and pain on emotion perception. J Clin Exp Neuropsychol.

[24] Fehm HL, Kern W, Peters A (2006). The selfish brain: competition for energy resources. Prog Brain Res.

[25] Bailey S (1999). American Diabetes Association Complete Guide to Diabetes.

[26] Demakis GJ, Harrison DW (1997). Relationships between verbal and nonverbal fluency measures: implications for assessment of executive functioning. Psychology Reports.

[27] Demaree HA, Harrison DW (1997). Physiological and neuropsychological correlates of hostility. Neuropsychologia.

[28] Donhoe RT, Benton D (1999). Blood glucose control and aggressiveness in females. Personality Individ Differ.

[29] Niaura R, Banks SM, Ward KD, Stoney CM, Spiro A, Aldwin CM, Landsberg L, Weiss ST (2000). Hostility and the metabolic syndrome in older males: the normative aging study. Psychosom Med.

[30] Raikkonen K, Matthews KA, Salomon K (2003). Hostility predicts metabolic syndrome risk factors in children and adolescents. Health Psychol.

[31] Bowers D, Coslett HB, Bauer RM, Speedie LJ, Heilman KM (1987). Comprehension of emotional prosody following unilateral hemispheric lesions: processing defect versus distraction defect. Neuropsychologia.

[32] Heilman KM, Bowers D, Speedie L, Coslett HB (1984). Comprehension of affective and nonaffective prosody. Neurology.

[33] Heilman KM, Scholes R, Watson RT (1975). Auditory affective agnosia. Disturbed comprehension of affective speech. J Neurol Neurosurg Psychiatry.

[34] Demaree HA, Everhart DE, Youngstrom EA, Harrison DW (2005). Brain lateralization of emotional processing: historical roots and a future incorporating “dominance.”. Behav Cognit Neurosci Rev.

[35] Foster PS, Drago V, Ferguson BJ, Harrison DW (2008). Cerebral moderation of cardiovascular functioning: a functional cerebral systems perspective. Clin Neurophysiol.

[36] Foster PS, Drago V, Webster DG, Harrison DW, Crucian GP, Heilman KM (2008). Emotional influences on spatial attention. Neuropsychology.

[37] Shenal BV, Harrison DW (2004). Dynamic lateralization: hostility, cardiovascular regulation, and tachistoscopic recognition. Int J Neurosci.

[38] Shenal BV, Harrison DW, Demaree HA (2003). The neuropsychology of depression: a literature review and preliminary model. Neuropsychol Rev.

[39] Harrison DW, Gorelczenko PM (1990). Functional asymmetry for facial affect perception in high and Low Hostile men and women. Int J Neurosci.

[40] Herridge ML, Harrison DW, Mollet GA, Shenal B (2004). Hostility and facial affect recognition: effects of a cold pressor stressor on accuracy and cardiovascular reactivity. Brain Cognit.

[41] Herridge ML, Harrison DW, Demaree HA (1997). Hostility, facial configuration, and bilateral asymmetry on Galvanic skin response. Psychobiology.

[42] Rhodes RD, Hu SR, Harrison DW (2010) Diminished right frontal capacity in high-hostiles: facial dystonia and cardiovascular reactivity. Manuscript submitted for publication

[43] Demaree HA, Higgins DA, Williamson J, Harrison DW (2002). Asymmetry in hand grip strength and fatigue in low-and high-hostile men. Int J Neurosci.

[44] Foster PS, Williamson JB, Harrison DW (2005). The Ruff Figural Fluency Test: heightened right frontal lobe delta activity as a function of performance. Arch Clin Neuropsychol.

[45] Holland AK, Catoe A, Smith A, Hardin J, Rutledge J, Carmona J, Harrison DW (2010). Lateralized differences in left hemisphere activation as a function of digestive distress: changes in verbal fluency performance and regulation of diastolic blood pressure before and after food ingestion. Archives of Clinical Neuropsychology.

[46] Holland AK, Newton SE, Hinson DW, Hardin J, Coe M, Harrison DW (2014). Physiological and behavioural indices of hostility: an extension of the capacity model to include exposure to affective stress and right lateralized motor stress. Later Asymmet Body Brain Cognit.

[47] Benton A, de Hamsher KS (1976). Multilingual aphasia examination.

[48] Harrison DW, Gorelczenko PM, Cook J (1990). Sex differences in the functional asymmetry for facial affect perception. Int J Neurosci.

[49] Higgins DA, Harrison DW (1999). Sex differences in the functional cerebral laterality of cardiovascular reactivity to speech and a cold pressor. Archives of Clinical Neuropsychology.

[50] Snyder KA, Harrison DW, Gorman WJ (1996). Auditory affect perception in a dichotic listening paradigm as a function of verbal fluency classification. Int J Neurosci.

[51] Helmer DC, Ragland DR, Syme SL (1991). Hostility and coronary artery disease. Am J Epidemiol.

[52] Larkin KT, Martin RR, McClain SE (2002). Cynical hostility and the accuracy of decoding facial expressions of emotions. J Behav Med.

[53] Contrada RJ, Jussim L (1992). What does the Cook–Medley hostility scale measure? In search of an adequate measurement model. J Appl Soc Psychol.

[54] Christensen AJ, Wiebe JS, Lawton WJ (1997). Cynical hostility, powerful others control expectancies, and patient adherence in hemodialysis. Psychosom Med.

[55] Coren S, Porac C, Duncan P (1979). A behaviorally validated self-report inventory to assess four types of lateral preference. J Clin Exp Neuropsychol.

[56] Lee DM, Weinert SE, Miller EE (2002). A study of forearm versus finger stick glucose monitoring. Diabetes Technol Ther.

[57] Lee GP, Meador KJ, Loring DW, Bradley KP (2002). Lateralized changes in autonomic arousal during emotional processing in patients with unilateral temporal lobe seizure onset. Int J Neurosci.

[58] Tieszen KL, New JP (2003). Alternative site for blood glucose testing: do patients prefer it?. Diabetes Med.

[59] Demers J, Kane MP, Bakst G, Busch RS, Hamilton RA (2003). Accuracy of home blood glucose monitors using forearm blood samples: freestyle versus One Touch Ultra. Am J Health-Syst Pharmacy.

[60] White, J., Braco, F., Malone, J. (2002). Comparison of the freestyle and the one touch ultra blood glucose monitoring system using capillary blood from the forearm. In: Presented at the 62nd scientific sessions of the America Diabetes Association, June, 15, San Francisco

[61] Brunner GA, Ellmerer M, Sendlhofer G, Wutte A, Trajanoski Z, Schaupp L, Quehenberger F, Wach P, Krejs GJ, Pieber TR (1998). Validation of home blood glucose meters with respect to clinical and analytical approaches. Diabetes Care.

[62] Nichols JH, Howard C, Loman K, Miller C, Nyberg D, Chan DW (1995). Laboratory and bedside evaluation of portable glucose meters. Am J Clin Pathol.

[63] Rheney CC, Kirk JK (2000). Performance of three blood glucose meters. Ann Pharmacother.

[64] Peled N, Wong D, Gwalani SL (2002). Comparison of glucose levels in capillary blood sample. Diabetes Technology Therapy.

[65] Jungheim K, Koschinksy T (2002). Glucose monitoring at the thenar: evaluation of upper dermal blood glucose kinetics during rapid systemic blood glucose changes. Hormonal Metabolic Research.

[66] Everhart DE, Harrison DW (2002). Heart rate and fluency performance among high- and low-anxious men following autonomic stress. Int J Neurosci.

[67] Johnstone B, Holland D, Larimone C, Groth-Marnat G (2000). Language and Academic Abilities. Neuropsychological assessment in clinical practice.

[68] Ruff RM, Light RH, Parker SB, Levin HS (1997). The psychological construct of word fluency. Brain Language.

[69] Baldo JV, Shimamura AP, Delis DC, Kramer J, Kaplan E (2001). Verbal and design fluency in patients with frontal lobe lesions. J Int Neuropsychol Soc.

[70] Tucha OW, Smely CW, Lange KW (1999). Verbal and figural fluency in patients with mass lesions of the left or right frontal lobes. J Clin Exp Neuropsychol.

[71] Ruff RM, Allen CC, Farrow CE, Niemann H, Wylie T (1994). Figural fluency: differential impairment in patients with left versus right frontal lobe lesions. Arch Clin Neuropsychol.

[72] Winer BJ (1971). Statistical principles in experimental design.

[73] Dembroski TM, Costa PT (1987). Coronary prone behavior: components of the type A pattern and hostility. J Pers.

[74] Eckhardt C, Norlander B, Deffenbacher J (2004). The assessment of anger and hostility: a critical review. Aggression and Violent Behavior.

[75] Holland AK, Carmona JE, Harrison DW (2012). An extension of the functional cerebral systems approach to hostility: a capacity model utilizing a dual concurrent task paradigm. J Clin Exp Neuropsychol.

[76] Shapiro PA, Sloan RP, Bagiella E, Kuhl JP, Anjilvel S, Mann JJ (2000). Cerebral activation, hostility, and cardiovascular control during mental stress. J Psychosom Res.

[77] Louis AG, Buchsbaum MS, Gillin JC, Wu J, Reynolds CA, Herrera DB (1992). The effect of Anxiety and hostility in silent mentation on localized cerebral glucose metabolism. Compr Psychiatry.

[78] Kinsbourne M (1980). Mapping a behavioral cerebral space. Int J Neurosci.

[79] Reuter-Lorenz PA, Kinsbourne M, Moscovitch M (1990). Hemispheric control of spatial attention. Brain Congnit.

[80] Selye, H. (1976). The stress of life (revised edition). McGraw-Hill Book Co, New York

[81] Dwyer DS (2002). Glucose metabolism in the brain.

[82] Jacobson AM, Samson JA, Weinger K, Ryan CM, Dwyer DS (2002). Diabetics, the brain, and behavior: Is there a biological mechanism underlying the association between diabetes and depression?. Glucose metabolism in the brain.

[83] Henderson DC, Ettinger ER, Dwyer DS (2002). Schizophrenia and diabetes. Glucose metabolism in the brain.

[84] McCall AL, Dwyer DS (2002). Diabetes mellitus and the central nervous system. Glucose metabolism in the brain.

[85] Woo E, Ma JT, Robinson JD, Yu YL (1988). Hyperglycemia is a stress response in acute stroke. Stroke.

[86] Barefoot JC, Dodge KA, Peterson BL, Dahlstrom WG, Williams RB (1989). The Cook–Medley hostility scale: item content and ability to predict survival. Psychosom Med.

[87] Guijarro ML, Hallet AJ, Miller TQ, Smith TW, Turner CW (1996). A meta analytic review of research on hostility and physical health. Psychol Bull.

[88] Chen CC, Lu FH, Wu JS, Chang CJ (2001). Correlation between serum lipid concentrations and psychological distress. Psychiatry Res.

[89] Schroeder EB, Chambless LE, Liao D, Prineas RJ, Evans GW, Rosamond WD, Heiss G (2005). Diabetes, glucose, insulin, and heart rate variability: the Atherosclerosis Risk in Communities (ARIC) study. Diabetes Care.

[90] Harrison DW (2015). Brain asymmetry and neural systems: foundations in clinical neuroscience and neuropsychology.

[91] Trajanoski Z, Brunner GA, Gfrerer RJ, Wach P, Peiber TR (1996). Accuracy of home blood glucose meters during hypoglycemia. Diabetes Care.

